# A pilot clinical assessment of biphasic asymmetric pulsed field ablation catheter for pulmonary vein isolation

**DOI:** 10.3389/fcvm.2024.1266195

**Published:** 2024-02-07

**Authors:** Bingwei Chen, Chang Lv, Yingjian Cui, Chengzhi Lu, Heng Cai, Zhixiao Xue, Xinyu Xu, Siying Su

**Affiliations:** ^1^ School of Biomedical Engineering and Technology, Tianjin Medical University, Tianjin, China; ^2^ Department of Cardiology, Tianjin First Central Hospital, Tianjin, China; ^3^ Department of Cardiology, Tianjin Medical University General Hospital, Tianjin, China; ^4^ Department of Research and Development, Tianjin Intelligent Health Medical Co., Ltd., Tianjin, China

**Keywords:** pulsed field ablation, pulmonary vein isolation, atrial fibrillation, biphasic asymmetric pulse, pilot clinical assessment

## Abstract

Pulsed field ablation (PFA) is a new treatment for atrial fibrillation (AF), and its selective ablation characteristics give it a significant advantage in treatment. In previous cellular and animal experiments, we have demonstrated that biphasic asymmetric pulses can be used to ablate myocardial tissue. However, small-scale clinical trials are needed to test whether this approach is safe and feasible before extensive clinical trials can be performed. Therefore, the purpose of this experiment is to determine the safety and feasibility of biphasic asymmetric pulses in patients with AF and is to lay the foundation for a larger clinical trial. Ablation was performed in 10 patients with AF using biphasic asymmetric pulses. Voltage mapping was performed before and after PFA operation to help us detect the change in the electrical voltage of the pulmonary veins (PV). 3-Dimensional mapping system showed continuous low potential in the ablation site, and pulmonary vein isolation (PVI) was achieved in all four PV of the patients. There were no recurrences, PV stenosis, or other serious adverse events during the 12 months follow-up. The results suggest that PFA using biphasic asymmetric waveforms for patients with AF is safe, durable, and effective and that a larger clinical trial could begin.

**Clinical Trial Registration:**

https://www.chictr.org.cn/, identifier, ChiCTR2100051894.

## Introduction

Atrial fibrillation (AF) is the most common sustained arrhythmia. Attacks of AF directly affect quality of life and increase mortality in patients (1, 2). Studies have shown that catheter ablation is an effective means to restore and maintain sinus rhythm in patients with AF (3, 4). Currently, catheter ablation procedures, such as radiofrequency ablation and cryoballoon ablation, are commonly used for the treatment of AF. Radiofrequency ablation applies high-frequency alternating current to cause myocardial tissue damage (5), while cryoballoon ablation relies on low-temperature energy to induce cellular necrosis through freezing (6). However, both techniques carry the risk of excessive damage and require control over the ablation energy, which may result in incomplete ablation or adverse effects on surrounding tissues and blood vessels.

Pulsed field ablation (PFA) is a non-thermal modality unlike every other ablation energy source. PFA is based on irreversible electroporation technology, which applies electric field intensity above the lethal electric field threshold for cell death to disrupt cell membranes and induce cell death. The lethal electric field threshold for cell death not only depends on tissue type but also on pulse parameters and other factors (7). Recent *in vitro* studies have only partially confirmed the tissue specificity of PFA. Apart from skeletal muscle (8), myocardial cells have a lower lethal electric field threshold for cell death, especially compared to esophageal smooth muscle cells and phrenic nerve cells. Myocardial cells are more susceptible to electroporation injury, with a lower threshold for cell death than other tissues (7, 9, 10). This selectivity is a fundamental cellular characteristic (11). Experimental results from different animal models suggest that PFA is a relatively safe ablation method, minimizing collateral damage to non-target tissues. While causing sufficient depth of myocardial ablation lesions, it does not harm important structures such as the esophagus (12, 13), phrenic nerve (14), and coronary arteries (15), and no histopathological changes have been observed (16). Imaging examinations of clinical trials of PFA also show a low incidence of complications such as phrenic nerve paralysis, esophageal injury, coronary artery spasm, and pulmonary vein stenosis after PFA (17, 18).

Based on previous experience in the application of irreversible electroporation for tumor ablation, our team has independently designed a biphasic asymmetric high-frequency pulsed electric field (PFA) system and a matching circular six-leaf catheter. We have conducted cell experiments and animal experiments (10, 19), continuously improving the pulse waveform and catheter parameters during the process, to develop a better ablation strategy. The results showed that PVI using this ablation model is safe, durable, and effective without causing damage to other tissues. But the feasibility of biphasic asymmetric pulses has not been proven in clinical practice. Thus, we conducted a pilot PULSED AF clinical trial (The Clinical Trial of Pulsed Field Ablation Therapy Apparatus; ChiCTR2100051894). This trial aims to demonstrate the safety and feasibility of biphasic asymmetric PFA in the treatment of AF patients and lay the foundation for subsequent larger-scale clinical trials.

## Methods

### Trial design

The Clinical Trial of Pulsed Field Ablation Therapy Apparatus (ChiCTR2100051894) clinical trial is a prospective, single-arm, safety and feasibility study of PFA conducted at Tianjin First Central Hospital. An independent clinical events committee adjudicated endpoint events. The study was funded and conducted by Tianjin Intelligent Health Co., Ltd., which is the manufacturer of the PFA instrument in this trial. All data have been entered in a study database, and the trials were approved by Chinese ethics committees and national regulatory agencies.

### Study population

The trial enrolled 10 patients aged 18–75 years with documented symptomatic persistent or paroxysmal AF. Patients should understand the procedure and voluntarily signed the informed consent. Detailed inclusion and exclusion criteria are provided in Supplementary Table S1.

### PFA system

The PFA system has 2 components: a pulse generator and a PFA ablation catheter with a 13-F steerable sheath (Tianjin Intelligent Health Co., Ltd., Tianjin, CHN).

**Figure 1:**
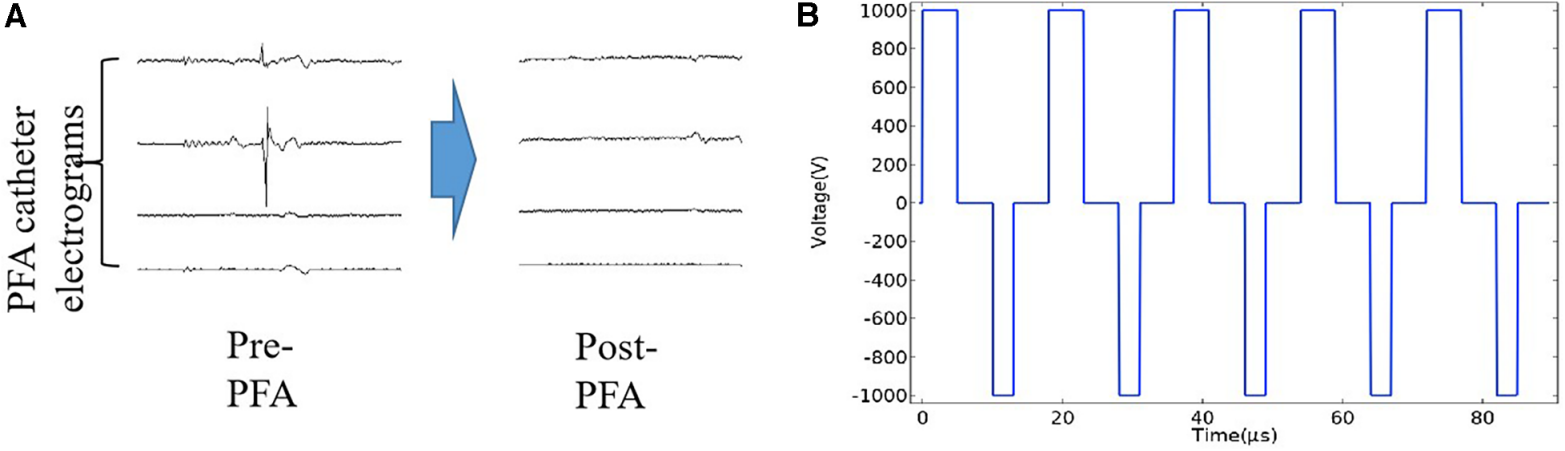
PV electrograms and pulse waveform diagram. (**A**) The PV electrograms can be obtained through the electrode of the PFA catheter frame. The PV potential is displayed immediately after the PFA application, showing the electrical isolation of the PV. (**B**) The pulsed field ablation mode used in this study was a biphasic asymmetric pulse, and the waveform diagram was generated using COMSOL simulation software.

The parameters, including the number of pulses, number of pulse groups, and pulse amplitude, could be set up on the generator. Energy is delivered through all of the electrodes in a proprietary sequence. Adjacent splines alternate as positive and negative electrodes and discharge simultaneously. Preclinical studies have optimized the therapeutic biphasic waveform, a structure that is a set of microsecond biphasic pulses that are synchronized with the heart rhythm (Figure 1B). All positive pulses applied in this study have a pulse width of 5 µs and negative pulses have a pulse width of 3 µs. The interval between two phase pulses is 5 µs, and each group of pulses consists of ten two-phase pulses. A complete discharge process includes ten groups of pulses. The pulses release 1,000 V microsecond pulses through the ECG synchronization signal.

**Figure 2:**
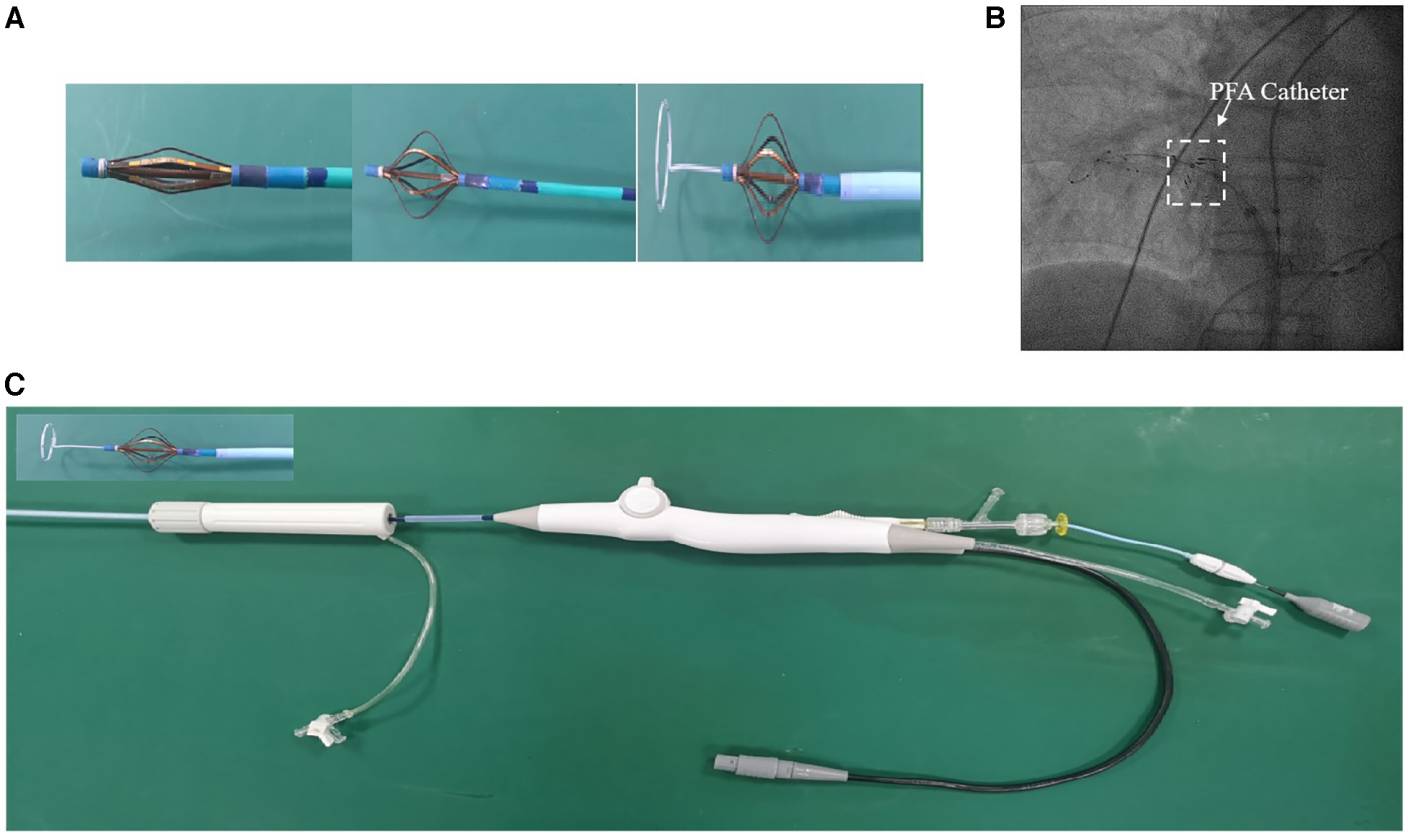
Pulsed field ablation catheter. (**A**) Appearance of the PFA catheter tip. From left to right, the catheter is closed, opened to basket position and carried with the mapping catheter. (**B**) Fluoroscopic image showing the state of the catheter when it is open, located in the pulmonary vein. The white dashed box indicates the location of the catheter. (**C**) The PFA catheter and its connecting parts include sheath, connecting wire and ECG mapping electrode.

The 10.5 F PFA catheter had six splines, there is one electrode with three markers on each spline (Figure 2). When fully deployed, the diameter of the distal portion is 32 mm, and the distance between adjacent electrodes is about 16 mm. The catheter is advanced over a guidewire to achieve circumferential contact of the frames with the target tissue. Ablative energy is delivered from all electrodes. The amplitude of the pulse and the number of pulses can be adjusted. Based on our previous experiments, we have utilized a positive pulse width of 5 µs, a negative pulse width of 3 µs, a pulse voltage of 1,000 V, and an approximate current of 10A.

### Procedural workflow

After signing the informed consent form, all patients' vital signs, blood counts, liver and kidney functions and ECG signals were tested preoperatively. The patient was placed in a supine position during the procedure, body electrodes were taped, and oxygen saturation monitoring was connected. The surgery was performed under local anesthesia, with the administration of analgesics during the procedure. After routine disinfection in the catheter room, a vascular sheath was placed by puncture in the right femoral vein. Heparin was administered intravenously without interruption during the procedure. A long guidewire is fed through the vascular sheath from the femoral vein into the superior vena cava. The septal puncture needle is delivered into the right atrium under the guidance of the guidewire. With the aid of x-ray fluoroscopy, an atrial septal puncture is performed, at which point the puncture needle enters the left atrium. Under fluoroscopy, contrast is rapidly pushed in to visualize the left atrial structure. A bendable sheath is then fed through the guide wire traction to perform a pulmonary venogram in order to visualize the pulmonary vein alignment. In this case, the catheter is delivered to the target ablation site. Ablation is performed with normal signal feedback from the angiogram and PFA instrument. The pulse voltage, pulse width, frequency and electrode distance of the PFA system used in this study can be adjusted to ensure the ablation effect. In addition, we performed voltage mapping of each patient's heart using Carto (Johnson & Johnson, America) before and after PFA to assess the ablation results. After the completion of each ablation, pacing was performed within the PV to demonstrate afferent and efferent block.

### Follow-up

Most patients experience a recurrence of arrhythmia within one year, with 15.3% of patients experiencing late recurrences (>12 months) (20). Based on the literature from most clinical trials on PFA, we limited the follow-up period to one year. All participants received clinical follow-up at 1 month, 3 months, 6 months and 12 months after the procedure. To evaluate the safety of PFA for AF, patients underwent routine physical examinations, 24-h ambulatory electrocardiogram monitoring and echocardiography at each follow-up visit. CT angiography was performed at the third-month follow-up. The feasibility was assessed by the incidence of atrial tachyarrhythmias and complications at the time of follow-up.

### Endpoint

The primary feasibility endpoint is the acute success rate of PV ablation, which is the probability of achieving PVI after the ablation procedure. Successful pulmonary vein ablation is defined as the pulmonary vein showing a low voltage (below 0.1 mV) on the voltage map. Voltage mapping was performed before and after PFA operation to help us detect the change in the electrical voltage of the PV.

The primary safety endpoint assessed in this trial was the incidence of device-related or procedure-related adverse events (AEs) such as death, stroke, phrenic nerve palsy, pericardial tamponade, atrioventricular fistula, and vascular complications at the puncture site during the 12-month postoperative follow-up.

The secondary feasibility endpoint was the proportion of patients remaining free of AF during the follow-up period. The guidance of *Clinical Study Designs for Surgical Ablation Devices for Treatment of Atrial Fibrillation* mentions that AF elimination (excluding medically induced arrhythmias) can be used to indicate the effectiveness of the procedure and not necessarily the return to normal sinus rhythm. Absence of AF should be defined as: no episodes of atrial fibrillation/atrial flutter (AFL)/atrial tachycardia (AT) lasting 30 s or longer on an ambulatory ECG or event recorder, or complete time recorded on a standard 12-lead ECG with no class I or III AADs. Regular ambulatory ECG monitoring is used as the preferred modality to assess effectiveness, although other modalities, such as standard 12-lead ECG, cross-phone recording, loop recorders, and event recorders may also be adequate. In this study, we chose to monitor patients with 24-h ambulatory electrocardiogram during the follow-up process. For both persistent and paroxysmal AF, the freedom from AF/AFL/AT for six months without of Class I or III AADs was the feasibility endpoint.

The secondary safety endpoint was defined as the rate of pulmonary vein stenosis occurring during the 12 months follow-up.

### Statistical analyses

Our experiment is a feasibility study without formal assumption check. Participants were followed up on the basis of consent to treatment. This technology was assessed for safety and efficacy endpoints according to the protocol. Results are descriptive statistics. The description of indicators will be calculated as mean, standard deviation, and percentage.

## Result

### Patients

Ten patients diagnosed with AF were enrolled between October 2021 and November 2021. Three of them had persistent AF and seven had paroxysmal AF. The selected patients were treated with PFA by physicians with extensive clinical experience. Three of the participants were male and the others were female. Baseline demographics are shown in Table 1 for the entire cohort. The participant had a mean age of 63.2 ± 7.2 years and mean left ventricular ejection fraction of 56.7 ± 7.4% with mean LA diameter of 34.8 ± 4.8 mm. All patients were on at least one antiarrhythmic drug.

**Table 1:** Baseline patient characteristics (*N* = 10).

Index	Number
Age, year	63.2 ± 7.2
Male	3 (30%)
LA diameter, mm	34.8 ± 4.8
LVEF, %	56.7 ± 7.4
CHA_2_DS_2_-VASc	2.3 ± 1.1
Hypertension	7 (70%)
Diabetes	5 (50%)
COPD	0 (0)
Dyslipidemia	0 (0)
CHD	2 (20%)
PCI	1 (10%)
Prior stroke	0 (0)
Antiarrhythmic medications	
Class I	5 (50%)
Class II	4 (40%)
Class III	4 (40%)
NOAC	10 (100%)

Values are mean ± SD or *n* (%).

CHA_2_DS_2_-VASc = congestive heart failure, hypertension, age ≥ 75 years, diabetes mellitus, stroke, vascular disease, age 65–74 years, sex category (female); CHD, coronary heart disease; COPD, chronic obstructive pulmonary disease; LA, left atrium; LVEF, left ventricular ejection fraction; NOAC, novel oral anticoagulants; PCI, percutaneous coronary intervention.

### Procedural characteristics

All 10 patients (100%) were successfully treated with PFA. The mean total operation time was 220.1 ± 47.2 min (Table 2). The mean fluoroscopy time was 35.1 ± 10.6 min. The average duration of PFA catheter stay in the body was 69.1 ± 8.3 min, and the time of PFA catheter stay in the body was defined as the time from the introduction of the ablation catheter to its removal from the body.

**Table 2:** Procedural characteristics (*N* = 10).

Index	Time (min)
Procedure time, min	220.1 ± 47.2
x-ray fluoroscopy time, min	35.1 ± 10.6
Catheter dwell time, min	69.1 ± 8.3
Total time of PV lesions per patient, min	
LSPV, min	1.94 ± 0.89
LIPV, min	2.3 ± 1.33
RSPV, min	2.02 ± 1.25
RIPV, min	2.4 ± 1.68

Values are mean ± SD.

LIPV, left inferior pulmonary veins; LSPV, left superior pulmonary veins; RIPV, right inferior pulmonary veins; RSPV, right superior pulmonary veins.

There were no complications associated with the PFA catheter, such as failure of placement, or thrombosis after catheter removal. No atrial or ventricular tachycardia, or significant repolarization abnormalities were found on 12-lead electrocardiograms. No patients had significant postoperative symptoms of muscle discomfort in the thoracic spine or upper extremities or motor nerve injury.

### Primary endpoint

**Figure 3:**
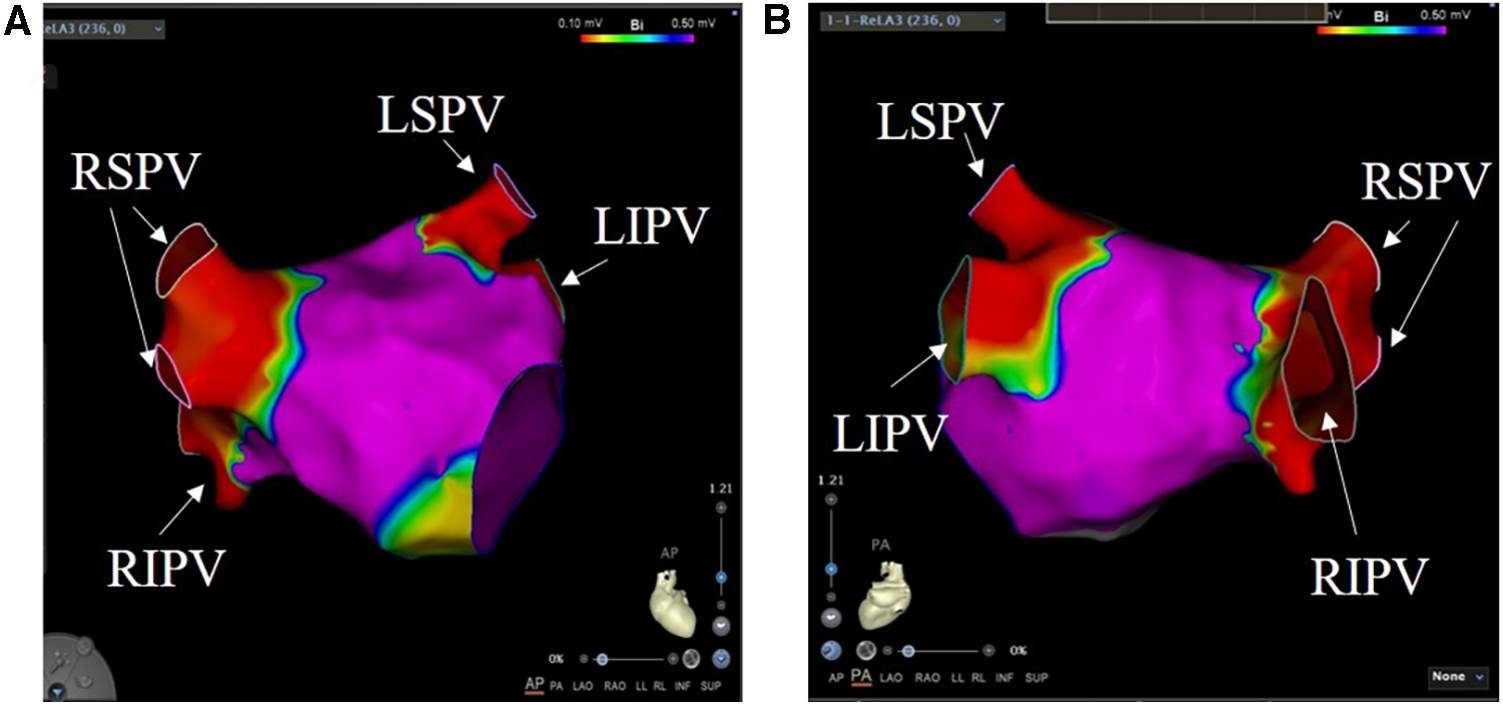
Pulmonary vein ablation. Voltage mapping was performed at the end of the index PFA procedure. The color scale of the bipolar voltage values is shown at the top of the picture: values above 0.5 mV are considered normal atrial tissue and depicted in purple. (**A**) is a view of the heart from the front of the body, and (**B**) is a view of the heart from the back of the body. RSPV, right superior pulmonary vein; RIPV, right inferior pulmonary vein; LSPV, left superior pulmonary vein; LIPV, left inferior pulmonary vein.

The primary feasibility endpoint of PVI was achieved in 100% of patients (Table 3). We also used lasso electrodes during the ablation procedure. When it confirmed there was no more potential, we performed further voltage mapping. The images indicate that the PV have been successfully isolated (Figure 3). In addition, we examined the patients' cardiac electrograms. It can be observed that the abnormal ECG signal has disappeared (Figure 1A). All 40 target PVs were successfully electrically isolated (PV show low voltage) with a mean discharge time of 8.66 min per patient. Because the ablation process requires accurate finding of the four PVs, it also requires the assistance of x-rays. Therefore, the catheter stays in the body for a long period of time, taking an average of 69.1 ± 8.3 min. On average, each PV was ablated three times. A single ablation was defined as a complete discharge process of the PFA system. Some of the repeat ablations were designed to achieve durable isolation, and the results showed that all PVs were acutely isolated in the first 1 or 2 applications.

**Table 3:** Primary feasibility endpoint (*N* = 10).

Item	Number
Acute PVI	10/10 (100)
AF elimination^a^	10/10 (100)
Number of ablations per PV	
LSPV	4.3 ± 2.49
LIPV	2.8 ± 1.75
RSPV	3.5 ± 1.17
RIPV	3.2 ± 1.54

Values are *n*/*N* (%).

^a^ AF elimination refers to no episodes of AF/atrial flutter (AFL)/atrial tachycardia (AT) lasting 30 s or longer on an ambulatory ECG or event recorder, or complete time recorded on a standard 12-lead ECG with no class I or III AADs. LIPV, left inferior pulmonary veins; LSPV, left superior pulmonary veins; RIPV, right inferior pulmonary veins; RSPV, right superior pulmonary veins.

Regarding the primary safety endpoint, we did not observe device-related or procedure-related major adverse events as death, pericardial tamponade/perforation, diaphragmatic paralysis, stroke or TIA, atrial-esophageal fistulas and vascular complications at the puncture site (Table 4).

**Table 4:** Primary safety endpoint (*N* = 10).

Symptom	Number			
	1 month	3 months	6 months	12 months
Death	0/10 (0)	0/10 (0)	0/10 (0)	0/10 (0)
Pericardial tamponade/perforation	0/10 (0)	0/10 (0)	0/10 (0)	0/10 (0)
Diaphragmatic paralysis	0/10 (0)	0/10 (0)	0/10 (0)	0/10 (0)
Stroke or TIA	0/10 (0)	0/10 (0)	0/10 (0)	0/10 (0)
Atrial-esophageal fistulas	0/10 (0)	0/10 (0)	0/10 (0)	0/10 (0)
Vascular complications at the puncture site	0/10 (0)	0/10 (0)	0/10 (0)	0/10 (0)

Values are *n*/*N* (%).

TIA, transient ischemic attack.

### Secondary endpoint

**Figure 4:**
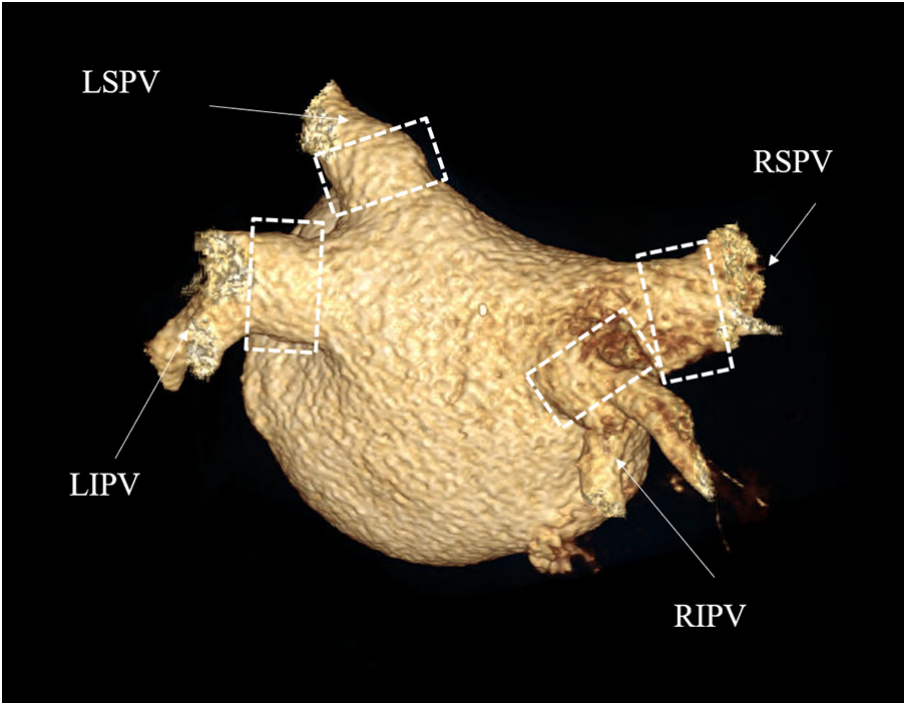
CTA of pulmonary veins. Postoperative 3D reconstruction images of the pulmonary veins. The four branches of the pulmonary veins were normal in size and direction, and no pulmonary vein stenosis occurred. The white dashed box is the location of the surgical ablation. RSPV, right superior pulmonary vein; RIPV, right inferior pulmonary vein; LSPV, left superior pulmonary vein; LIPV, left inferior pulmonary vein.

Regarding the secondary feasibility endpoint, no patient experienced a recurrence during the 12-month follow-up. Regarding the secondary safety endpoint, CT angiography was performed at the third-month follow-up, and the results showed no pulmonary vein stenosis in all patients. There were no differences in the PV and their branches compared to pre-operation, and they functioned normally (Table 5 and Figure 4).

**Table 5:** Secondary endpoint (*N* = 10).

Symptom	Number			
	1 month	3 months	6 months	12 months
Recurrence of AF	0/10 (0)	0/10 (0)	0/10 (0)	0/10 (0)
Pulmonary vein stenosis	0/10 (0)	0/10 (0)	0/10 (0)	0/10 (0)
Stroke or TIA	0/10 (0)	0/10 (0)	0/10 (0)	0/10 (0)

Values are *n*/*N* (%).

TIA, transient ischemic attack.

## Discussion

### Biphasic asymmetric pulse and other pulse parameters

Lavee et al. (21) first applied the irreversible electroporation technique, namely PFA, to cardiac ablation, with a pulse pattern of 1,000–1,500 V unidirectional DC pulse sequence and a single pulse duration of 100 µs. However, unidirectional DC pulses may cause local and systemic muscle contractions, leading to electrode displacement, altering the electric field distribution, and affecting the treatment efficacy (22). At the same time, unidirectional DC pulses may also cause electrochemical reactions on the electrode surface, leading to gas embolism. Therefore, in subsequent clinical applications, it is necessary to maintain general anesthesia and use high doses of muscle relaxants (23, 24). Subsequently, a high-frequency biphasic pulse discharge pattern was proposed, which effectively reduced pain and muscle contraction (25) and decrease or completely eliminate the formation of related gas bubbles (24). However, due to the canceling effect of biphasic pulses, they require higher pulse amplitudes to achieve similar ablation effects as single-phase pulses. This canceling effect depends on the delay and pulse duration between the positive and negative pulses, and it can be partially explained by auxiliary discharges, depending on the medium (26). We speculate that the inhibitory effect of negative pulses on ion flow in the solution leads to a decrease in conductivity and current density, thereby reducing the ablation efficiency. On this basis, a new pulse discharge pattern was proposed, using a biphasic asymmetric pulse in the ablation field. Results from cell experiments showed that the biphasic asymmetric pulse significantly reduced the lethal electric field threshold compared to biphasic symmetric pulses by 59% (27), achieving the same ablation effect with lower ablation energy. Currently, the biphasic pulse pattern has been widely applied in clinical trials of PFA, allowing for surgery under local anesthesia with good efficacy and safety observed in postoperative follow-up (28). Although mild muscle contractions were observed in some *in vivo* experiments during biphasic pulse therapy (29), the degree of muscle contraction was significantly reduced compared to single-phase pulses. One possible explanation is that the relative size of the electrodes and the treated animals may affect the extent of muscle contraction (24). In our previous animal experiments and in this study, no significant muscle contractions were observed.

In the process of pulsed field ablation therapy, the application of high-voltage pulses generates electrical stimulation on nerve fibers in and around the treatment area, and even at a distance, causing muscle contractions and pain. The minimum electric field strength required to induce nerve fiber stimulation is known as the electrical stimulation threshold. As the pulse width increases, the lethal electric field threshold gradually decreases, and the stimulation threshold also decreases, but at a much faster rate (30). Single-phase pulses are more efficient in penetrating and killing cells, while biphasic pulses can reduce or avoid muscle twitching (31). Both thresholds depend on the waveform configuration (24). We aim for our asymmetric biphasic waveform to minimize the increase in the lethal electric field threshold, while preventing muscle twitching by not reaching the electrical stimulation threshold in the distal nerve fibers. Due to the alternation of positive and negative pulses and hardware limitations, the negative pulse width is set at 3 µs, which is currently the shortest pulse width achievable by our team. With increasing frequency, muscle contractions significantly decrease (24), and the duration of the positive pulse is greater than that of the negative pulse, hence the positive pulse width is set at 5 µs. Additionally, we do not consider a 40% difference between the negative and positive pulse widths to be insignificant. The purpose of the asymmetric pulses is to lower the lethal electric field threshold. Previous experiments have shown that the 2 µs single-phase high-frequency pulse is nearly equivalent to the 50 µs single-phase high-frequency pulse in terms of the lethal electric field threshold (27). Adding a phase delay between positive and negative pulse can not only protect the PFA system, but also provides therapeutic advantages, as the phase delay allows the cell transmembrane voltage discharge and polarity reversal not to be forced immediately (25). We have set a 5 µs inter-phase delay between the positive and negative pulses. Most pulse schemes utilize a single-phase pulse sequence of 100 µs (30, 32) possibly due to the pulse duration being between 80 and 100 µs, which maximizes energy output (33). Based on the above experimental results, we have configured a single pulse sequence as 5 sets of asymmetric biphasic pulses, comprising 10 positive and negative pulses, with a total pulse sequence duration of 90 µs. Although the mechanism of irreversible electroporation is non-thermal, there is still a possibility of thermal damage due to the proximity of tissue and electrodes. Pulsed cycles can maintain lower tissue temperatures (34), and we have added a certain delay between each pulse sequence to reduce the overall risk of thermal damage. In the early treatment plan, 100 pulse discharges were performed at each position, and studies have shown that the volume of damage produced by 40–200 pulse discharges is equal (35). Therefore, each discharge process of ours includes 10 sets of pulse sequences, that is, 50 sets of asymmetric biphasic pulses. Based on the current PFA system, our preliminary cell experiments and animal studies have demonstrated that a pulse amplitude of 1,000 V can achieve safe and durable PVI.

### PFA technique and pre-experimentation

PFA is a more controlled form of energy delivery that uses multiple brief DC pulses that can be delivered through multiple electrodes in a matter of seconds. Adjacent myocardial cell membranes are disrupted to form nanoscale pores, cell membrane permeability is increased, and cell contents leak. This leads to immediate necrosis or apoptosis (36). PFA has the following features: (1) the electric field intensity threshold required for cardiomyocyte death is the lowest of all tissue types; (2) because the mechanism of cell death is non-thermal, the unexpected damage is lower than that of thermal energy. It has been shown that even when PFA is placed directly on these structures, it does not cause PV stenosis, phrenic nerve injury, or esophageal injury (13, 14). In clinical therapy, PVI by radiofrequency ablation is achieved by point-by-point ablation, which in turn creates a circumferential ablation zone. This model requires further mapping and ablation. In contrast, PFA allows the direct formation of a closed-loop isolated region, avoiding the problem of RF ablation and therefore substantially reducing ablation time.

Our team conducted extensive preparation work in preclinical research. In cellular experiments, we established a single-cell system and a monolayer cell system. PFA was performed on H9C2 myocardial cells and A7r5 smooth muscle cells, demonstrating that H9C2 cells were more sensitive to PFA. We discussed the target points and various pulse parameters of PFA for the treatment of AF, and ultimately determined the bipolar asymmetrical pulse discharge mode (10). In animal experiments, we first used a four-valve catheter combined with a pulse generator to verify the durability and safety of PVI in preclinical research. It effectively reduced the energy threshold for ablation and reduced muscle contractions. Pathological results showed that the ablation area of myocardial cells was replaced by fibroblasts, without damage to adjacent tissues (19). In subsequent animal experiments, we applied an improved six-valve circular catheter combined with a pulse generator and completed PFA in 15 dogs. We also performed anatomical examinations at different time points to explain the reasons for short-term recurrence and self-healing of AF, as well as discussed the apoptotic mechanisms of myocardial cells after ablation (37). We applied the experience accumulated in the early stage to clinical trials. This experimental PFA clinical trial evaluated the effectiveness and safety of the PFA system in patients. Follow-up trials and longer follow-up periods are planned.

### Safety and feasibility of PFA

PFA is safe in the treatment of AF. There were no further major safety events during follow-up, including no atrial esophageal fistula, phrenic nerve palsy, PV stenosis, or stroke. A distinctive characteristic of PFA is its ability to “selectively” ablate myocardial tissue while preserving nearby structures, like the esophagus and phrenic nerve. This is because the energy threshold required to induce necrosis in cardiomyocytes during this process appears to be the lowest of all tissue types. Ekanem et al. (38) studied the safety of PFA in the treatment of AF. Their retrospective survey, which included all 24 clinical centers using PFA catheters after regulatory approval, obtained evidence of the safety of PFA for the treatment of AF. Cochet et al. (18) studied the safety of PFA by evaluating damage to the esophagus, and no esophageal lesions were found in all patients during the postoperative and three-month follow-up periods. Füting et al. (39) evaluated the phrenic nerve separately before and after cardiac ablation and found that PFA did not cause damage to the phrenic nerve. Numerous animal studies have shown that PFA also reduces the risk of PV stenosis. In these preclinical experiments, catheter ablation produced durable lesions in the atria while no PV stenosis was observed (40, 41).

The occurrence of PV reconnection after radiofrequency or cryogenic PVI remains common, regardless of whether the patient is taking the relevant medication. Additional ablation targeting the area of reconnection can improve the outcome of the procedure, but this approach increases the time and cost of the procedure (42). But this rarely happens with PFA. In this study, we evaluated the feasibility of pulsed electric field ablation for the electrical isolation of PVs in patients with paroxysmal or persistent AF. It was found that in 10 patients, 40 (100%) PVs were acutely isolated during the procedure. There was no recurrence of atrial fibrillation at our 12-month follow-up.

Reddy et al. (43) treated 66 patients with AF with biphasic PFA. The results showed that PVI was achieved in all patients during the procedure and no patient developed arrhythmias during the one-year follow-up. In addition, Reddy et al. (44) found in another study that PVI alone is considered inadequate for many patients with persistent AF and that left atrial posterior wall (LAPW) assisted ablation may improve prognosis. This provides a new way of research for the treatment of AF.

Because PFA delivers each pulse sequence within a single cardiac cycle, the energy application time is very short compared to conventional thermal catheter ablation. In contrast, cold/heat ablation requires continuous application to reach tissue temperature, thus causing irreparable damage to cardiac tissue. Therefore, efficiency will be another potential critical advantage of PFA (45).

### Current progress of PFA clinical trials

In 2019, a large number of clinical trials of PFA for AF have been conducted abroad. A clinical trial on the safety and effectiveness of pulse field therapy for AF showed that PFA was effective in 78% of AF patients estimated at 1 year. The major complication rate was low at 1.9%, with no esophageal injury or pulmonary vein stenosis (17). These results are consistent with our research findings, indicating that pulse field therapy can reduce collateral damage to non-target tissues and achieve good treatment outcomes. Another clinical trial treated drug-refractory AF patients (150 paroxysmal and 150 persistent cases) with PFA. The results showed that PFA was effective in 66.2% of paroxysmal AF patients and 55.1% of persistent AF patients within one year. The major adverse event rate was 0.7% (46). Although our study included 3 cases of persistent AF patients, the small number of cases led to a lack of statistical significance. Further large-scale clinical trials will make comparisons in this regard. In a clinical trial comparing PFA with conventional thermal ablation for treating paroxysmal AF, PFA showed no inferiority to conventional thermal ablation in terms of avoiding immediate isolation failure, repeat ablation, recurrence rate of arrhythmias, and incidence of device-related serious adverse events within one year (47). PFA, as a relatively novel ablation method, has the potential to further improve its effectiveness and safety with improved surgical techniques and combined auxiliary methods.

The field of catheters that match PFA is also constantly evolving. Clinical trials of catheter ablation for AF that can switch between radiofrequency and PFA showed an estimated freedom from arrhythmias of about 78% for paroxysmal and persistent AF patients within one year. The improved waveform treatment achieved a freedom from arrhythmias of 84.8% for patients with persistent AF (48), although these results are related to the relatively small number of patients using this waveform, they are also exciting. In a multicenter clinical trial of a new integrated circuit bipolar PFA catheter with a three-dimensional electroanatomic mapping system, this integrated system can map and track lesion locations, reduce unnecessary energy delivery, and reduce fluoroscopy time, demonstrating strong effectiveness and safety (28).

We did not detect any recurrences of atrial fibrillation during the follow-up period of our experiment, which seems somewhat incredible, but it is possible for a pilot clinical trial with only ten patients. The results of this trial only represent the treatment effect among these ten patients and cannot prove the superiority of our system as it has no statistical significance. The fact that three patients with persistent atrial fibrillation did not have recurrences one year after ablation keeps us optimistic about subsequent clinical trials, but also due to the limit of the sample size, it cannot be taken as evidence. The large-scale clinical trials and one-year postoperative follow-up we plan to carry out afterwards are the objective evaluations of the treatment effect and safety of our PFA system, and this pilot clinical trial has provided effective evidence and reference for the formulation of subsequent clinical research plans.

### Limitations

Further *in vivo* experiments are needed to study and validate the reasons behind the biphasic asymmetric pulses. The aim of this study was to investigate the safety and feasibility of using biphasic asymmetric pulse to achieve PVI for AF. Therefore, the sample size of this study is small and future larger trials are needed to confirm our findings. They were performed by experienced operators and larger multicenter studies are needed to assess their generalizability. Given the invasive nature of remapping studies, we did not perform remapping. Therefore, it was not demonstrated whether PV reconnection occurred during the follow-up period.

## Conclusions

In this human pilot trial, biphasic asymmetric PFA using catheters could safely and feasibility treat patients with persistent and paroxysmal AF, achieving immediate and long-term isolation of the PVs with no serious adverse events during long-term follow-up. Pulmonary vein isolation is safe and durable, therefore provides the basis for a large prospective study of arrhythmia control.

## Data Availability

The original contributions presented in the study are included in the article/Supplementary Material, further inquiries can be directed to the corresponding authors.
