# Supplementary material for: A pilot clinical assessment of biphasic asymmetric pulsed field ablation catheter for pulmonary vein isolation
Source: Front Cardiovasc Med. 2024 Feb 7;11:1266195. doi: 10.3389/fcvm.2024.1266195 (PMC10879394; doi:10.3389/fcvm.2024.1266195)
Supplement: Supplementary file 1 [file Table1.docx]

**Supplementary material**

| **Table 1 Inclusion criteria for participate with paroxysmal atrial fibrillation.** |
| --- |
| 1. Male or female between the ages of 18 - 80 years; 2. Patients diagnosed with symptomatic atrial fibrillation:   At least 1 ECG or rhythm monitoring documented AF episode within 12 months prior to enrollment;   1. Refractory to at least one class I or III AAD. (Refractory defined as not effective or not tolerated); 2. Willingness, ability, and commitment to participate in baseline and follow-up evaluations for the full length of the study; 3. Willingness and ability to give an informed consent. |
| AAD= anti-arrhythmic drugs; ECG= electrocardiogram; |

| **Table 2 Exclusion criteria for participate with paroxysmal atrial fibrillation.** |
| --- |
| 1. In the opinion of the investigator, any known contraindication to an atrial ablation, TEE, or anticoagulation. Including but not limited to the identification of any atrial thrombus or evidence of sepsis; 2. Continuous AF lasting longer than 12-months; 3. History of previous left atrial ablation or surgical treatment for AF/AFL/AT; 4. AF secondary to electrolyte imbalance, active thyroid disease, or any other reversible or non-cardiac cause; 5. Structural heart disease as described below:    1. Left ventricular ejection fraction (LVEF) < 40% based on most recent TTE;    2. Left atrial size > 55 mm (parasternal long axis view) documented within 6-months of screening;    3. NYHA Class III or IV heart failure documented within the previous 12-months;    4. An implanted pacemaker or ICD;    5. Previous cardiac surgery, ventriculotomy, or atriotomy (excluding atriotomy for CABG);    6. Previous cardiac valvular surgical or percutaneous procedure, or prosthetic valve;    7. Interatrial baffle, closure device, patch, or PFO occlude;    8. Presence of a left atrial appendage occlusion device;    9. Presence of any pulmonary vein stenting devices;    10. Coronary artery bypass graft (CABG) or PTCA procedure within six months prior to procedure;    11. Unstable angina or ongoing myocardial ischemia;    12. Myocardial infarction within the previous six months prior to procedure;    13. Moderate or severe mitral insufficiency or stenosis based on most recent TTE;    14. Atrial myxoma;    15. Significant congenital anomaly; 6. BMI > 40; 7. Any previous history of cryoglobulinemia; 8. History of blood clotting or bleeding disease; 9. History of severe COPD requiring steroid use in the previous 12-months; 10. History of severe sleep apnea (AHI > 30) not currently treated with a CPAP machine or other mechanical device; 11. Stroke or TIA within the last year; 12. Pregnant or lactating (current or anticipated during study follow-up; 13. Current enrollment in any other study protocol where testing or results from that study may interfere with the procedure or outcome measurements for this study; 14. Any other conditions such as, mental illness, addictive disease, terminal illness with a life expectancy of less than two years, extensive travel away from the research center that may lead to non-compliance with the protocol procedure or follow up. |
| BMI= body mass index; COPD= chronic obstructive pulmonary diseases; CPAP= continuous positive airway pressure; ICD= implantable cardioverter-defibrillator; PFO= patent foramen ovale; PTCA= percutaneous transluminal coronary angioplasty; TIA= transient ischemia attack; TTE= trans-thoracic echocardiography. |
